# Multi-disciplinary strategy to optimize irrigation efficiency in irrigated agriculture

**DOI:** 10.1038/s41598-024-61372-0

**Published:** 2024-05-19

**Authors:** Ayman Batisha

**Affiliations:** 1 Environment and Climate Change Research Institute, National Water Research Center, Cairo, Egypt; 2 Council of Future Studies and Risk Management, Academy of Scientific Research and Technology (ASRT), Ministry of Scientific Research, Cairo, Egypt

**Keywords:** Irrigation equilibrium indicators (IEIs), Climate, water, food, and energy (CWFE) nexus, Sustainable irrigated agriculture (SIA), Global goals (GGs), Efficiency, Water organizations, Climate sciences, Environmental sciences, Environmental social sciences, Hydrology, Energy science and technology, Engineering

## Abstract

Equilibrium among water, food, energy, and climate actions is necessary for life to exist, quality, and sustainability. This article explored how to ensure sustainability, and equilibrium in the irrigation processes by proposing irrigation equilibrium indicators (IEIs) for sustainable irrigated agriculture (SIA). The primary purpose of IEIs is to achieve a state of sustainable climate and environmental balance. The pressures driving agriculture and irrigation professionals to enhance the irrigation scheme performance are tremendous in all agricultural communities. Monitoring, assessment, and improvement of agriculture practices and irrigation schemes for enhancing the Climate, water, food, and energy (CWFE) nexus is a must. As an auspicious climate action, IEIs were developed to enhance the irrigation scheme’s efficiency, within the scope of SIA. Subsequently, water, agricultural, food, and energy productivity could be optimized. Then, the appropriate equilibrium indicators could identify the actual performance of the CWFE nexus as a whole and the performance of each component. The effective irrigation scheme is the backbone of SIA. IEIs could measure the degree of achieving the overall and specific objectives and designated irrigation processes. The ultimate measure of equilibrium is optimizing sustainable agricultural yields and productivity, ensuring environmental balance, strengthening life quality, and maximizing economic returns.

## Introduction

Water is the main bio element for life sustainability. If the water represents the blood, then the irrigation schemes and drainage practices represent the arteries, and veins respectively of a healthy life and sustainable human civilization. Sustainable irrigated agriculture (SIA) combines climate, water, and energy to sustain, intensify, and improve food, feed, fiber, and fuel production. Environmental, social, economic, technical, and management objectives of irrigated agriculture and its associated irrigation scheme should be identified. The overall goal of the sustainable irrigated agriculture (SIA) project is to enhance agricultural production and productivity through better management and control of irrigation water. Implied in these goals is the need to optimize crop water productivity, water investment efficiency^1^, and unconventional water resources^2^. The parameters are interrelated and could be applied to characterize the overall performance of the sustainable irrigated agriculture (SIA) system.

The pressures driving irrigation professionals to enhance the irrigation scheme performance are tremendous and especially in many low and middle-income nations of the world. The permanent reasons are the rapidly rising water stresses and population versus a relatively small cultivable land area, the increased expectation of rural dwellers resulting from the man-made and natural crises the growing mobility of people through immigration, and ultimately the increased dependence on river irrigation. Hazards correlated to sustainable irrigated agriculture (SIA) include climatological, atmospheric, hydrologic, biological, chemical, anthropogenic, natural, human-induced, and/or technological hazards. Global, regional, national, and local, disasters should also be considered. Nowadays, more catastrophic events such as international wars and conflicts (Gaza–Israel war, Russo-Ukrainian war)^3^, the proxy wars, the new cold war, the new superpower clash, the plunge in Sino-US relations, boiling points, political tumult, hardline politics, provoking acts, and global epidemics cause a more negative impact and pressure on all irrigation, water, food, energy, and agriculture professionals [e.g.,^4–6^]. The importance of developing irrigation equilibrium indicators (IEIs) in optimizing productivity, ensuring environmental balance, and maximizing economic returns in sustainable agriculture practices was highlighted.

Sustainable irrigation practice is a cross-action to facilitate fulfilling Global Goals (GGs). Sustainable irrigation could directly and positively impact the majority of (GGs), (e.g., Climate action, clean water, no hunger, no poverty, economic growth, sustainable communities, infrastructure, responsible consumption, peace, and justice…). Climate change, poor water delivery, and low irrigation application efficiency may lead to some unfavorable effects resulting in lower yield per unit (P.U.) of area and (P.U.) of freshwater, less total area irrigated, and detrimental environmental effects, as well as lower returns from the irrigated crops. Principally, Sustainable irrigation could be considered an effective climate action (CA) to adapt to water stress. Climate Actions (CAs), adequate predictable water supplies to meet crop needs, and water savings to be freed up for agricultural and other uses could be considered measures of sustainable future achievements.

**Figure 1:**
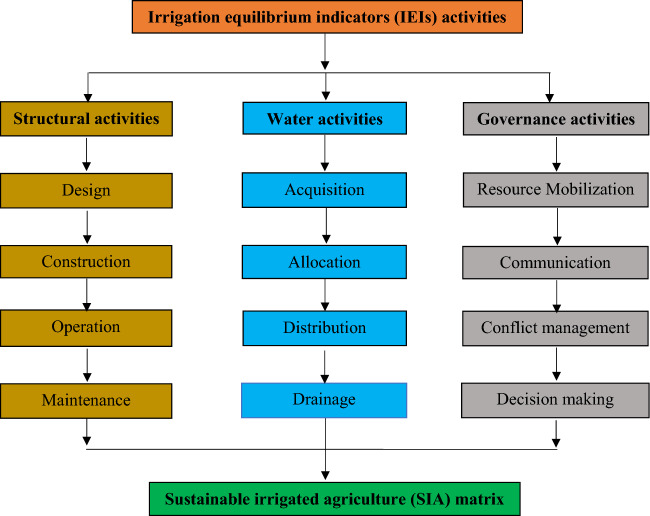
Analytical framework for Irrigation equilibrium indicators (IEIs).

Sustainable irrigated agriculture (SIA) focuses on the wise utilization of irrigation water. Water should be efficiently allocated to the plant root zone when required without waste and in adequate quantities. Control over water and its proper management for agricultural and other purposes are necessitated at all levels in the irrigation schemes. The sustainable irrigated agriculture (SIA) project components could be designed into groups of related functions and development options. There are three major components of irrigation water management activities. The water activities focus on water obtaining adequate and assured supplies (acquisition), scheduling (allocation), utilization at the exact place at the appropriate time (distribution), and prevention and removal of excess (drainage). Irrigation scheme performance relies not only on water management but on all irrigation scheme components including hydraulic structures, machines, information, stakeholder participation, and other inputs. The structural (hardware) activities focus on the provision of control (design and construction to capture and provide safe direction), operation (to achieve timely releases and adjustments in proper, quantities and elevations), and maintenance (sustaining the capability to provide scarce resources). The governance (software) activities focus on the actions of water organizations to precisely manage the scheme, (mobilizing resources, communicating the plans, managing conflict, and making decisions) within collective opinion, and carefully managing water resources. These three major focuses of irrigation water management activities, along with the four sub-divisions of each, form an equilibrium management matrix shown in Fig. 1.

The stakeholders should be more mindful that they have enough water (adequacy), that get their fair share (equity), and that comes when they need it (reliability). The stakeholders should be also more mindful that the man-made and natural resources base relative to erosion, water logging, and salinity is stable. These considerations of productivity, adequacy, equity, reliability, and stability are the primary criteria upon which sustainable irrigated agriculture (SIA) is judged by water organizations. Field construction and rehabilitation could represent physical and engineering activities that involve and incorporate hydraulic structural replacement (HSR), preventive maintenance, and irrigation improvement techniques sub-components. Human-based activities could incorporate professional development, training, and research and development which stress capacity building, either to conduct water organizations' routine work efficiently or to undertake research programs to solve specific problems. Conducting all these actions to maximize agricultural production and deliver the freshwater to the farm.

The assessment, monitoring, and improving needs for climate, water, food, and energy (CWFE) nexus are complex [e.g.,^7,8^]. The defined components could be contemplated as individual sectors in their own rights with stated objectives, human needs, training requirements, construction activities, and achievable outputs. To further discipline matters, the individual components should have their implementation departments within the Water organizations. Then, the appropriate equilibrium indicators could identify the actual performance of the CWFE nexus as a whole and the performance of each component. This study can be seen as the first step in coming up with meaningful equilibrium indicators for the CWFE nexus. It could be followed by others to validate and apply the concepts outlined. This would require further collaboration with representatives of all CWFE nexus components to facilitate collecting necessary data.

## Methods

Sustainable irrigated agriculture (SIA) should consider all issues related to socioeconomic, climate, environment, energy, irrigation, and agricultural practices. SIA mainly depends on freshwater resources, good governance, hydraulic structures, farmers’ organizations, law institutions, engineers, and efficient human resources. The optimal irrigation water utilization through the application of the right amounts of water necessary for optimal plant growth is the key to achieving SIA. Perhaps at the farm level, the primary management objective is to maximize the farmers' own profit. For the nation at large, the governance objectives should emphasize strengthening environmental and climate indicators. To evaluate the performance of sustainable irrigated agriculture (SIA) in meeting its objectives, continuous monitoring of the system status, inputs, and outputs is required. Collected data should be carefully examined and analyzed with systematic feedback to delineate means and ways of improving the system’s performance.

### Impact of efficiency on yield and agricultural production

The agricultural process goal is to optimize the grown crop amount (P.U.) of resource for a specified time. The agricultural efficiency could be determined as the ratio between the actual to the expected yield, i.e. the ratio between actual and expected crop quantity (P.U.) of resource (land, water, energy …etc.) for a specified time (season). The process starts by identifying Sustainable irrigated agriculture (SIA) project goals and objectives. It would be nice if management objectives were set to specify the desired level of system performance. For example, the goal of increasing agricultural productivity may establish yield levels that should be attained. The agricultural “Yield” is the harvest, crops, and earnings generated by an agricultural process over a particular period.

### Irrigation efficiency

**Figure 2:**
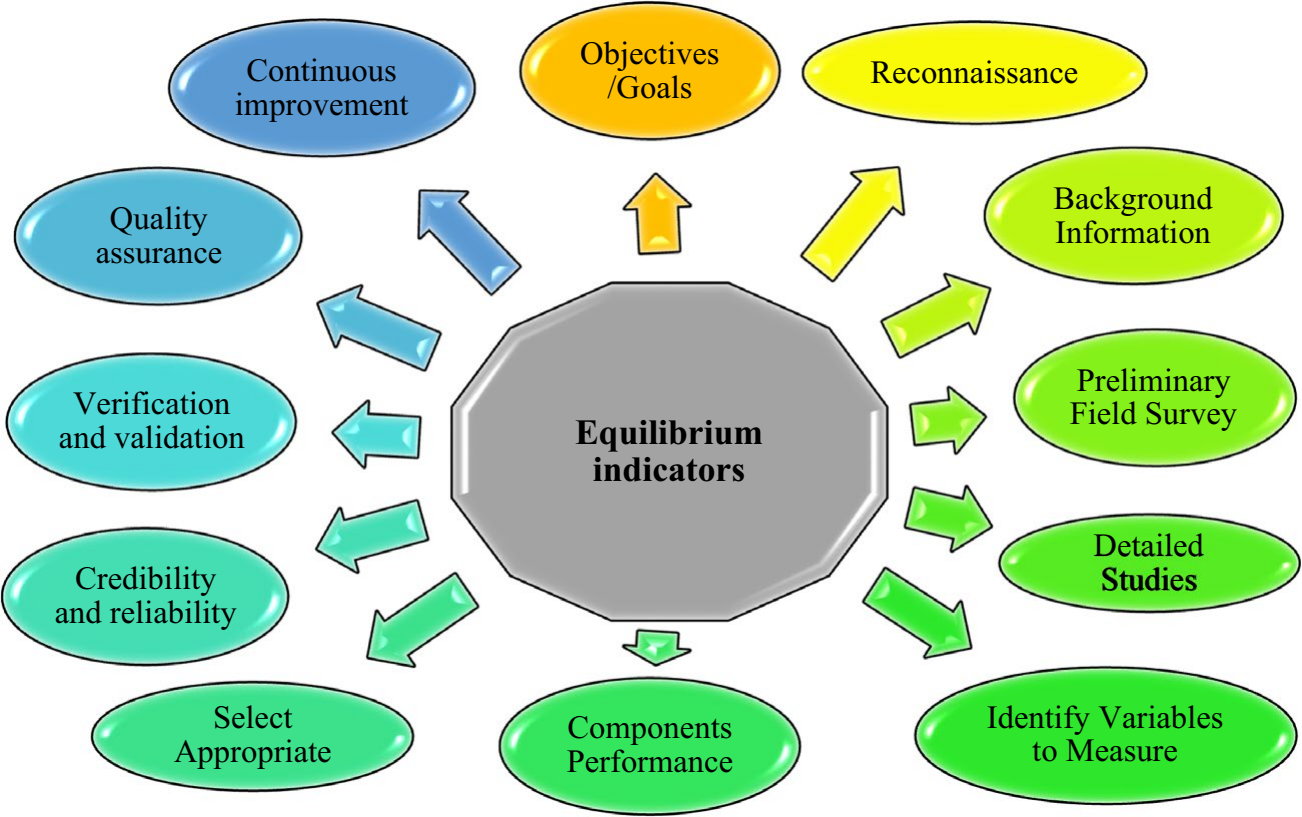
Methodology of design key equilibrium indicators.

Many scholars proposed indicators to assess irrigation efficiency, irrigated agriculture, and water use efficiencies. [e.g.,^9–15^]. The scope of work was defined, designed, and briefed as follows: Reviewing appropriate documents and literature for background information; organizing field trips to project sites for data collection, discussions, and performance assessment; Exploring the vision of climate, water, food, and energy leaders, directors, officers, scholars, researchers, engineers, and project teams for expectable nexus outputs; Considering appropriate Climate Actions (CAs) and adequate water supplies for the farmers as the measures for agricultural development goal achievement; Outlining procedures to arrive at the equilibrium indicators in a report format that could be computerized for data collection and updating; Designing nexus key equilibrium indicators as shown in Fig. 2 for the nexus components and investigating their relationships to the nexus as a whole.

### The mathematical process

The typical crop-water production function could represent agricultural yield for a specified time (season). The agricultural yield could be plotted as a function of the total applied water quantity. The function starts with a relatively high slope indicating that water is efficiently used to increase production at low levels of irrigation. When the applied water levels increase, the slope diminishes. At the maximum yield, the function will have zero slope (horizontal line). After that, additional increments of applied water tend to decrease the yield due to decreased aeration and gas transfer within the plant root zone. Commonly, the mathematical relationship between agricultural production (crop/yield) and irrigation scheme efficiency (η_I_) could be determined as a sigmoid function—continuously increasing function—(“S”-shaped curve).

### Efficiency of irrigation water

The agricultural yield is a percent of the maximum potential output which could be obtained when an irrigation efficiency of 100%. The three significant locations of the agrarian yield are characterized by the following:

- The first region—the low-efficiency phase—is where efficiency changes will affect slightly the yield.
- The second region—the medium-efficiency phase—is where efficiency changes affect strongly the yield.
- The third region—the high-efficiency phase—is where efficiency changes affect slightly the yield.

Irrigation efficiency could be determined as the ratio between the required Water quantity for beneficial use (P.U.) area (W_r_) to the Water quantity delivered and applied (P.U.) area (W_d_). The irrigation efficiency (η_I_) can be written as:

1 $$\eta_{{\text{I}}} = \left[ {\left( {{\text{W}}_{{\text{r}}} } \right)/\left( {{\text{W}}_{{\text{d}}} } \right)} \right]$$

Irrigated areas under most projects are limited by the available water quantities (W_t_). The potential irrigated area (I_a_) is given in general by:

2 $${\text{I}}_{{\text{a}}} = \left( {\left( {{\text{W}}_{{\text{t}}} } \right)/\left( {{\text{W}}_{{\text{d}}} } \right)} \right]$$

Equations (1) and (2) could be combined to show that, for a specified total available water quantity (W_t_) and a recommended beneficially applied water quantity (W_r_), the irrigated area is directly correlated to the irrigation efficiency.

3 $${\text{I}}_{{\text{a}}} = \left[ {\left( {{\text{W}}_{{\text{t}}} } \right) \, \left( {\eta_{{\text{I}}} } \right)/\left( {{\text{W}}_{{\text{r}}} } \right)} \right]$$

The agricultural output (P.U.) of freshwater (Y_w_) can be correlated to the agricultural output (P.U.) of area (Y_a_) as follows:

4 $${\text{Y}}_{{\text{w}}} = \left[ {\left( {{\text{Y}}_{{\text{a}}} } \right)/\left( {{\text{W}}_{{\text{d}}} } \right)} \right]$$

Equations (1) and (4) could be combined to express the agricultural output (P.U.) of freshwater concerning the irrigation efficiency

5 $${\text{Y}}_{{\text{w}}} = \left[ {\left( {{\text{Y}}_{{\text{a}}} } \right)\left( {\eta_{{\text{I}}} } \right)/\left( {{\text{W}}_{{\text{r}}} } \right)} \right]$$

Equations (3) and (5) show that a decrease in the irrigation efficiency will cause a decrease in both the potential irrigated area I_a_ and the agricultural output (P.U.) of freshwater Y_w_. Lower irrigation efficiency may also contribute to various unfavorable environmental impacts such as: leaching nutrients from the plant root zones, soil erosion and deterioration of soil structure, and rising groundwater levels increasing the possibility for drainage problems and salinity hazards. The necessity for optimal irrigation water efficiency is obvious.

### The overall climate, water, food, and energy (CWFE) nexus efficiency

**Figure 3:**
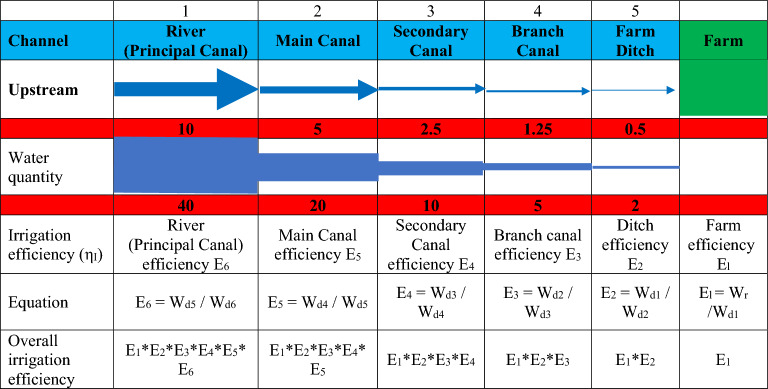
Overall irrigation efficiency for an irrigation scheme with five sections.

When evaluating the performance of the climate, water, food, and energy (CWFE) nexus, it is often useful to assess the effectiveness of all system components. This allows us to identify components that are not performing well. The irrigation scheme can be schematically described by a package of successive sections as displayed in Fig. 3 where each section is characterized by the freshwater quantity which is delivered as compared with water losses. For the *i*th segment of the irrigation scheme, the segment efficiency could be defined as:

6 $$\eta_{{{\text{sec}}}} = {\text{ W}}_{{\text{d}}} \left( {i - {1}} \right)/{\text{W}}_{{\text{d}}} \left( i \right)$$

and the water losses within the section are given by:

7 $${\text{W}}_{{\text{L}}} = {\text{ W}}_{{\text{d}}} \left( i \right) \, - {\text{ W}}_{{\text{d}}} \left( {i - {1}} \right)$$

where W_d_(*i*) is the quantity of water allocated into the *i*th section. It could be noted that for the first section (*i* = 1), the irrigated field, W_d_(*i*-1) = W_rl_ which represents the beneficially required amount of water for agricultural production.

Hence: the farm efficiency E_l_ = W_r_ /W_d1_

the farm ditch efficiency E_2_ = W_d1_/W_d2_

the branch canal efficiency E_3_ = W_d2_/W_d3_

and therefore, the overall branch canal efficiency E_1,3_ is given by

8 $$\begin{aligned} {\text{E}}_{{{1},{3}}} & = {\text{ W}}_{{{\text{rl}}}} /{\text{Wd3}} \\ {\text{E}}_{{{1},{3}}} & = \, \left( {{\text{W}}_{{{\text{rl}}}} ,{\text{ W}}_{{{\text{d1}}}} } \right)\left( {{\text{W}}_{{{\text{d1}}}} /{\text{W}}_{{{\text{d2}}}} } \right)\left( {{\text{W}}_{{{\text{d2}}}} /{\text{W}}_{{{\text{d3}}}} } \right) \\ {\text{E}}_{{{1},{3}}} & = \, \left( {{\text{E}}_{{1}} } \right)\left( {{\text{E}}_{{2}} } \right)\left( {{\text{E}}_{{3}} } \right) \\ \end{aligned}$$

**Figure 4:**
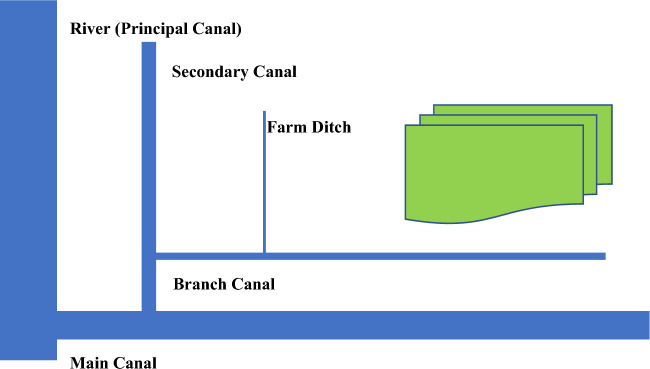
Overall irrigation efficiency for an irrigation scheme.

According to Fig. 4, the overall irrigation scheme efficiency is equal to the multiplication of the efficiencies of its components.

9 $${\text{E}}_{{{1},{\text{ n}}}} = \, \left( {{\text{E}}_{{\text{l}}} } \right) \, \left( {{\text{E}}_{{2}} } \right) \, \left( {{\text{E}}_{{3}} } \right) \, \left( {{\text{E}}_{{{\text{n}} - {1}}} } \right) \, \left( {{\text{E}}_{{\text{n}}} } \right)$$

When evaluating the seasonal irrigation efficiency, the quantity of water involved is seasonal. The need for improvement can be defined in the section where the efficiency is lowest. With on-farm irrigation improvement, agricultural efficiency is correlated to individual irrigation. Usually this is accomplished by analyzing the freshwater distribution profile under the farm area.

### Climate actions (CAs) and crop yield

The obtained yield is the major indicator for defining agricultural Climate Action (CA) performance. Food efficiency could be a tool for achieving carbon and climate targets [e.g.,^16^]. Although the yield of the irrigated field is considerably correlated to other factors apart from climate (irrigation, fertilizer, cultivation, socioeconomics … etc.), it can be correlated to the Climate Action (CA) performance, especially for known and given levels of the other components. The evaluation of the Climate Action (CA) performance of the overall agricultural season with respect to yield is associated with the simultaneous analysis of the following terms:

The ratio of agricultural yield before and after Climate Action (CA) (P.U.) of freshwater

10 $${\text{R}}_{{\text{w}}} = \left( {{\text{Y}}_{{{\text{aa}}}} } \right)/\left( {{\text{Y}}_{{{\text{ea}}}} } \right) = \left( {{\text{Y}}_{{\text{w}}} } \right)/\left( {^{\prime } {\text{Y}}_{{\text{w}}} } \right)$$

The ratio of agricultural yield before and after Climate Action (CA) (P.U.) of area

11 $${\text{R}}_{{\text{a}}} = \left( {{\text{Y}}_{{{\text{aa}}}} } \right)/\left( {{\text{Y}}_{{{\text{ea}}}} } \right) = \left( {{\text{Y}}_{{\text{a}}} } \right)/\left( {^{\prime } {\text{Y}}_{{\text{a}}} } \right)$$

The ratio of agricultural yield before and after Climate Action (CA) (P.U.) of energy

12 $${\text{R}}_{{\text{e}}} = \, \left( {{\text{Y}}_{{{\text{aa}}}} } \right) \, / \, \left( {{\text{Y}}_{{{\text{ea}}}} } \right) \, = \, \left( {{\text{Y}}_{{\text{e}}} } \right) \, / \, \left( {^{\prime } {\text{Y}}_{{\text{e}}} } \right)$$

The expected yield (P.U.) of irrigation water, (P.U.) of area, and (P.U.) of energy is correlated to these variable sources.

the average yield normally obtained in the studied area or other areas with similar conditions.

13 $$^{\prime } {\text{Y}}_{{\text{a}}} = {1}/{\text{n}}\left( {{\text{Y}}_{{\text{a}}} } \right)_{{\text{j}}}$$

in which (Y_a_)_j_ is the yield (P.U.) of area for the *j*th season, and n is the number of seasons observed. The expected yield (P.U.) of freshwater regarding the applied water quantity in the *j*th season (W_i_) is given by:

14 $$^{\prime } {\text{Y}}_{{\text{w}}} = {\text{I}}/n\left[ {\left( {{\text{Y}}_{{\text{a}}} } \right)_{{\text{j}}} /{\text{Wi}}} \right]$$

the optimal yield obtained from local experiments or by adaptation of relevant data from other sources.

Evaluation of the overall irrigation season is related to the simultaneous analysis of both R_w_, R_a_ and R_e_. The various possible combinations are as follows:

- All R_w_, R_a_ and R_e_ have high values. Actual yields are high for both units of water, area, and energy. The overall irrigation season is regarded satisfactory, and the condition is considered the most desirable.
- R_a_ is low while R_w_ is high. Actual yields are lower than expected, although the yield (P.U.) of irrigation water is high. Consequently, the water application is rather efficient, but the total depth of water application is too low resulting in a lower yield.
- R_a_ is high while R_w_ is low. Actual yields are higher than expected, but actual yields (P.U.) of freshwater are low. The higher yield is a result of sufficient water but inefficient application. Additional relatively large quantities of water result in relatively small increase in yield; however, those yields are higher than expected.
- All R_w_, R_a_ and R_e_ are low. Large quantities of freshwater and energy result in yields lower than expected, and freshwater and energy are used inefficiently. Results are most unfavorable, and yields indicate poor irrigation and energy performance.

### Equilibrium indicators

Efficient control and application of water would free extra amounts to be utilized for extending the cultivated area or to allow shifts to new crop varieties that increase the grower returns. The following derived indicators reflect the effectiveness of using freshwater in economic or absolute terms. Such indicators could be applied at the National or at the command level. Comparison of the values of these macro indicators before and after the execution of the sustainable irrigated agriculture (SIA) project would reflect the success of the project in meeting its goals.

15 $${\text{Equilibrium}}\,{\text{indicator}}\,\left( {1} \right)\,{\text{reflects}}\,{\text{the}}\,{\text{worth}}\,{\text{of}}\,{\text{agricultural}}\,{\text{production}}\,\left( {{\text{P}}.{\text{U}}.} \right)\,{\text{of}}\,{\text{freshwater}}\,\left( {\text{or energy}} \right)\,{\text{used}}\left( {\$ /{\text{m}}^{{3}} } \right) = \left( {{\text{value}}\,{\text{of}}\,{\text{the}}\,{\text{agrarian}}\,{\text{production}}/{\text{water}}\,{\text{quantity}}\left( {{\text{or}}\,{\text{energy}}} \right)\,{\text{used}}} \right)$$

16 $${\text{Equilibrium}}\,{\text{indicator}}\left( {2} \right){\text{reflects}}\,{\text{the}}\,{\text{extent}}\,{\text{of}}\,{\text{the}}\,{\text{agrarian}}\,{\text{area}}\,{\text{irrigated}}\left( {{\text{P}}.{\text{U}}.} \right)\,{\text{of}}\,{\text{freshwater}},\,\left( {{\text{hectare}}/{\text{m}}^{{3}} } \right)\left( {\text{or energy}} \right) = \left( {{\text{Total}}\,{\text{land}}\,{\text{area}}\,{\text{in}}\,{\text{production}}/{\text{water }}\left( {{\text{or}}\,{\text{energy}}} \right){\text{quantity}}\, {\text{used}}} \right)$$

17 $$\begin{aligned} & {\text{Equilibrium}}\,{\text{indicator}}\left( {3} \right){\text{reflects}}\,{\text{the}}\,{\text{overall}}\,{\text{use}}\,{\text{efficiency}}\,{\text{regarding}}\,{\text{meeting}}\,{\text{the}}\,{\text{crop}}\,{\text{watering}}\left( {{\text{or}}\,{\text{energy}}} \right){\text{requirements}}\,\% \\ & = \left( {{\text{Water}}\left( {{\text{or}}\,{\text{energy}}} \right)\,{\text{demand}}\,{\text{for}}\,{\text{crop}}\,{\text{production/water}}\left( {{\text{or}}\,{\text{energy}}} \right){\text{quantity}}\,{\text{used}}} \right) \\ \end{aligned}$$

Each of the previous indicators has its own merits and limitations.

In the usage of such indicators over a period, problems associated with shifts in the cropping regime and prices due to changes in the world economy would be encountered. Again, agricultural production is a function of the effectiveness of Climate Actions (CAs) and other agricultural inputs (water, land, energy, …). The usage of the previous three indicators would be not very practical before all Sustainable irrigated agriculture (SIA) activities are completed. The strength of using such indicators will be observed when they are applied to two similar areas, one with clear Sustainable irrigated agriculture (SIA) project involvement and the other without Sustainable irrigated agriculture (SIA) involvement.

## Results

The climate, water, food, and energy (CWFE) nexus are a multi-disciplinary hub of greatest importance to sustainable irrigated agriculture (SIA). Several concepts and definitions have been outlined in this study concerning possible indicators to assess the performance of all components of the climate, water, food, and energy (CWFE) nexus and the integrated project as a whole. The CWFE nexus could be represented as a cluster of overlapping components, each having its objectives to generate an array of outputs.

The designed climate, water, food, and energy (CWFE) nexus could represent a promising innovation that contributes to strengthening the water organization's capabilities in planning, designing, operating, and maintaining the irrigation scheme. The objectives and functions of some designed climate, water, food, and energy (CWFE) nexus components are very much related and there should be room for greater interaction between such components. The designed climate, water, food, and energy (CWFE) nexus emphasizes some components each of which has its specific objectives which is linked "somehow" to the nexus overall objectives. Because of the magnitude and importance of the designed climate, water, food, and energy (CWFE) nexus, the assessment and monitoring needs are vital and complex.

### Hierarchy of climate, water, food, and energy (CWFE) nexus

**Figure 5:**
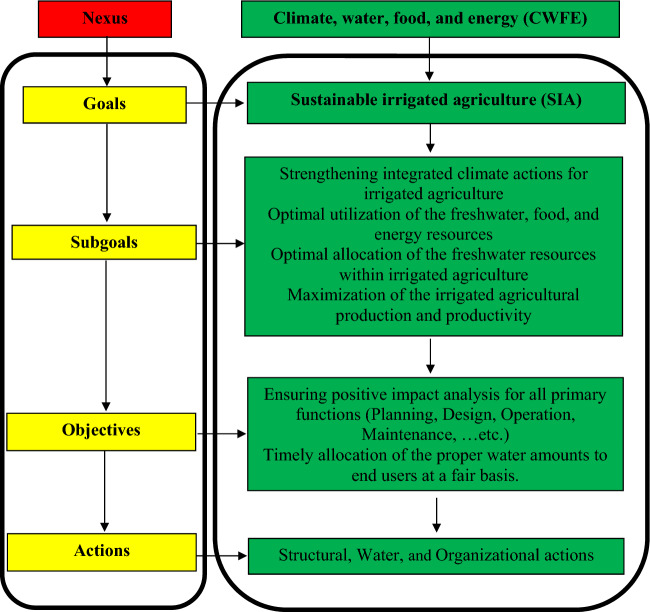
Objective hierarchy of climate, water, food, and energy (CWFE) nexus.

The CWFE nexus has an array of strategic milestones arranged in four hierarchical levels of goal, subgoal, objectives, and actions as outlined in Fig. 5. The study mission is to develop equilibrium indicators that would tell how successful the components and the integrated nexus package are in achieving the stated objectives. The overall goal is to sustain irrigated agriculture; and increase agricultural production and productivity. The designed nexus subgoal is to enhance water control and use efficiency. Performance indicators could reflect progress toward meeting physical, financial, or institutional achievements, regarding designated objectives and outputs of the climate, water, food, and energy (CWFE) nexus. Construction of hydraulic structures and conveyance works, expenditure in commodities and training, and the reinforcement of water users’ associations or Irrigation Advisory Services are respective examples of physical, financial, and institutional achievement.

### Water (climate/food/energy) organizations

The strengthening of all primary functions could be demonstrated by the flow of research results applicable to water organizations' irrigation primary functions (planning, design, operation, and maintenance), upgrading water organizations' staff capabilities and effectiveness to execute the previous functions. To that end, the vision of the following components should be clear: water research activities, professional development activities, and irrigation improvement.

For some designed climate, water, food, and energy (CWFE) nexus components, it is practicable to trace the impact at the nexus goal, subgoal, and objective levels by quantifying variables correlated to crop production or water control and reliability (irrigation improvement, preventive maintenance, hydraulic structures rehabilitation and replacement, information technology, artificial intelligence, planning studies, and mathematical models). Other supporting components including (professional development, training, capacity building, nexus preparation, nexus feasibility, advanced nexus (climate, water, food, and energy) research, futurology and sustainability studies, survey and mapping, and advanced technologies) directly strengthen designed climate, water, food, and energy (CWFE) nexus at the objective level.

At the subgoal level of improving the operation freshwater distribution efficiency for agricultural irrigation and other uses, the impact of a few components can be directly traced to the climate, water, food, and energy (CWFE) nexus objectives. This subgoal objective is measured by the truth that farmers have adequate and predictable water supplies and water is freed up for agricultural and other uses. To that end, the contribution of the planning studies; mathematical models; information technology; artificial intelligence; preventive maintenance; hydraulic structures rehabilitation; water research, professional development; and irrigation improvement; are should be clear through the strengthening performance of irrigation equilibrium indicators (IEIs).

The function of professional development concerning the training activities conducted by all designed climate, water, food, and energy (CWFE) nexus components. The task of information technology, artificial intelligence, planning studies, and mathematical models is utilizing real-time data provided by advanced technologies for better operation of the allocation system. Contribution of advanced water and nexus research, futurology, and sustainability studies to problems and issues of concern to other CWFE nexus components. Interaction between preventive maintenance, hydraulic structures rehabilitation, and replacement, to set optimal schedules of maintenance versus replacement.

### The overall CWFE nexus components

The following section is devoted to tracing components to perform the overall CWFE nexus objectives concerning the prescribed four levels. Equilibrium indicators of the CWFE nexus components should focus on improvement impact analysis. So, Equilibrium indicators concentrate on how to strengthen the water organizations’ capability and capacity for planning, design, operation, and maintenance either directly or indirectly as given below:

### Planning and design

The strengthening of Planning and Design items could be demonstrated by the ability of water organizations to implement projects and programs that are well designed, carefully analyzed, qualitatively implemented, and adequately financed. To this effect, the mission of the following components should be clear: planning studies; mathematical models; survey and mapping; project preparation; project feasibility; and irrigation improvement.

### Operation

The strengthening of operation items could be demonstrated by smart utilization of the hydraulic structures and improved control of the freshwater distribution resulting in timely allocation of the proper amounts to end users on a fair basis. To this effect, the mission of the following components should be clear: planning studies; mathematical models; information technology; artificial intelligence (AI); and irrigation improvement.

### Maintenance

The strengthening of maintenance items could be demonstrated by having an irrigation scheme that is maintained to prolong infrastructure investment. To that end, the contribution of the following components should be clear: preventive maintenance; hydraulic structures rehabilitation and replacement.

### Irrigation equilibrium indicators (IEIs)

Equilibrium indicators ideally should gauge an impact, have limited numbers (few), be objectively measured, have high validity, and have their data easily collected. The focus is on improvement programs of hydraulic structures, water techniques (irrigation schemes and drainage practices), and water governance organizations, as follows:

### Hydraulic structures improvement program

- Quantity of freshwater delivered to users at the sample canal end.
- Quantity of water utilized within a given canal command.
- Number of complaints from water users over a year.
- Number of days of irrigation compensation following off periods in the rotations over a year.

### Water (irrigation schemes and drainage practices) improvement program

- Yield is measured at the threshold, middle, and tail ends of a small demonstration irrigation canal (mesqa).
- Adequacy of freshwater (delivery of required amounts of water) is measured at the threshold, middle, and end of the small demonstration canal (mesqa).
- Progress in improvement program execution manifested by the sample area fully improved each year.
- Amount of water spilled unused to the drainage system at the tail end of sample canals.
- The volume of complaints from water users along the canal, and disputes between neighboring directorates (the downstream directorates are not receiving their full share).
- Number of key points along the irrigation schemes and drainage practices covered in yearly flow issued reports.
- Percentage (regarding total area and different agricultural seasons) of high-quality aerial photography and various kinds of maps suitable for planning, design, and monitoring purposes of irrigation schemes and drainage practices.
- Percentage (regarding of total area and different agricultural seasons) of photo interpretation methods to provide necessary data for other climate, water, food, and energy (CWFE) nexus components and departments within the water organizations.

### Water organizations improvement program

- Percentage of professionals within the water organizations who are familiar with the advanced technologies, and their capabilities to use mathematical models, information technology, and artificial intelligence.
- Percentage of study reports that illustrate the merits of the advanced technologies used over current practices in predicting inflow to channels and waterways, operating the hydraulic structures, and water distributing via the irrigation scheme.
- Equilibrium indicator matrix that incorporates relevance, status, and utilization of all advanced technologies, (100% for best performance).
- Number of studies (hydrological, technical, feasibility, environmental impact assessment (EIA), risk assessment, …) completed each year for projects included in the water organizations five-year plan.
- Number of studies (hydrological, technical, feasibility, environmental impact assessment (EIA), risk assessment, …) completed for other components of climate, water, food, and energy (CWFE) nexus.
- Professional Development concerning growth in professional course enrollment, number, duration, and their scientific levels within water organizations.
- Percentage of professional courses in advanced technologies utilization to other courses within water organizations.
- Satisfaction of trainees, trainee supervisors, and instructors about training environment and professional courses.
- A number of acceptable technical reports produced each year addressing problems and concerns related to the water organizations or the climate, water, food, and energy (CWFE) nexus at large.
- Number, Category, Rank, Percentile, and impact of technical papers published in referred journals or scientific conferences relevant to the water organizations or the climate, water, food, and energy (CWFE) nexus at large.

### Optimize CWFE nexus synergies using IEIs

The Irrigation equilibrium indicators (IEIs) could be applied to Optimize climate, water, food, and energy (CWFE) nexus synergies. The equilibrium indicators could be measured by the impact traced through the objective hierarchy achievement displayed in Fig. 5 by the integrated components of the climate, water, food, and energy (CWFE) nexus. At the goal level, the primary measure of objective fulfillment is the sustainable future yield. The influence of the integrated climate, water, food, and energy (CWFE) nexus could be reflected through the global indicator given by Eq. (15). The influence of the Improvement Project could be directly measured at that level through equilibrium indicators. The different components directly affect the subgoals of better operating efficiency and control, leading to indirectly contributing to achieving the main goal. These components lead to improved delivery (adequacy, reliability, and uniformity) along the canal and mesqa systems which would automatically cause the yield increase.

The yield, revenue, and agricultural production could be used as indicators of sustainable irrigated agriculture (SIA) (climate, water, food, and energy (CWFE) nexus goal), and water investment efficiency in irrigation (CWFE subgoal). Capacity building and training should be linked to the CWFE purpose of strengthening the water organizations’ capabilities for planning, design, operation, and maintenance. Then, sustainability and future requirements concerning the effectiveness of CWFE equilibrium indicators should be strengthened.

A major equilibrium indicator of the effectiveness of sustainable irrigated agriculture is that of crop yield. The yield (P.U.) area in main crops (wheat, rice, maize, cotton, sugar cane) should be acceptable for farmers and investors. Farmers' desire to increase yields (P.U.) area should depend on high prerequisite levels of all agricultural process inputs, abundance of freshwater, and sophisticated, and sustainable practices for crop husbandry. The agricultural yield should ultimately achieve strategic objectives at the local, national, and global levels.

Water application (Irrigation) system efficiency is often the focus in the quest for increased yield (P.U.) of freshwater and for agricultural water conservation. The concept of efficiency is reflected in the three global indicators given by Eqs. (15), (16) and (17) expressing respectively the worth of agricultural production (P.U.) of freshwater used, the extent of the area in production (P.U.) of freshwater used, and the actual water requirement relative to the quantity of water allocated (for a unit area). The climate, water, food, and energy (CWFE) nexus impact on a given canal command could be observed through the usage of the foregoing three indicators.

## Discussion

The synergy between sustainable development keystones (e.g., water, energy, food …) and goals (e.g., no poverty, no hunger, good health, clean water, clean energy, Peace …) is a must to ensure a sustainable and illustrious future. The nexus concept has recently been employed to highlight the interconnected relationship governing water, food, and other sustainable development keystones. Water, food, energy, and climate are must to sustain life and quality of life nationally and globally. In the context of water-food-energy nexus, recent scholars’ interests and objectives included practices of sustainable agriculture^17^, African countries^18^, Drylands^19^, rooftop gardening in urban areas^20^, cities^21^, resilience and sustainability^22^, land use^23^, solar panels^24^, simultaneously, the probable and unforeseeable consequences of floating solar panels on water reservoirs should be assessed^25^. Some other scholars have extended water-food-energy nexus to involve other various keystones, (e.g., ecology^26^, forests^27^, Carbon dioxide (CO_2_) emissions^28^).

In another context, a meta-approach perspective for water as the bio of life and a keystone of global goals (GGs) was introduced^29^. A holistic water framework composed of Demographics, Ecological, Environmental, Political, Economic, Social, and Technological (DEEPEST) drivers was presented. The components are Demographics (e.g., population growth, densities, and migration, …), Ecological (e.g., ecosystems, aquatic ecosystems, and freshwater ecosystems, …), Environmental (e.g., climate change, drought, desertification, irrigation, and drainage …), Political (e.g., water policy, political stability, war, conflict, and peace, …), Economic (e.g., circular economy, global markets, agricultural economics, hydro-economic models, commercial water demand, …), Social (e.g., education, communications, awareness, poverty, and well-being, …), and Technological (e.g., emerging technologies, nanotechnology, biotechnology, and digital technologies, …).

### Water at time of war, river fragments, and climate change

Politicizing or militarizing water, food, and agriculture-sensitive issues should be absolutely prevented. Military actions related to armed conflict have a severe negative impact on water infrastructure and water resources. Consequently, most Global Goals (GGs) could be very difficult or impossible to fulfill. Water sustainability-related harms in future armed conflicts should thoroughly be prevented^30^. As a recent example, the Russia–Ukraine military conflict negatively impacted water reservoirs, dams, and water infrastructure^31^. The damage to the water infrastructure (irrigation, water supply, flood protection, drainage, and sanitation sector) is inclusively evaluated to be US$2.6 billion. Food security, food crises, and agrifood systems should be reconsidered due to the war^32^. The key strategic adverse impact is on agriculture^33,34^. Food production was negatively impacted by geopolitical crises, and weather events^35^, the Russia–Ukraine armed conflict^36,37^.

Shaping future plans for sustainable freshwater supply in the conflict regions and ensuring water infrastructure and systems rehabilitation were proposed^38^. The impacts of other armed conflicts on freshwater resources were reported (e.g., freshwater Lake Chad, Africa^39^, water deficit and drought, Central America^40^. Transboundary water cooperation and collaborations are necessitated to build peace and resilience^41^ and sustainable development^42^. Policy actions should enhance environmental solutions and remove or reduce barriers^43^.

Obviously, more pressures would be generated from nature, global change, climate change, and/or man-made hazards necessitating more serious and immediate actions. Multiple dams on the world's great rivers (e.g., Amazon, Mekong, and Congo), adversely impact hydrology, ecosystem, and river sediments^44^. Dam construction fragments the river regime, and dam operation regulates discharge, and impacts hydrological, nutrient, and sediment processes^45^. Considerable irrigation schemes have become inactive and failed to be effective for the development in sub-Saharan Africa (SSA)^46^.

### Sustainable irrigation and agriculture

The positive impact of perennial irrigation and its related drainage works on soil, people, and welfare has been emphasized since the nineteenth century^47^. As old as the Pharaohs, Egyptian irrigation practice is an art, and culture that made the Nile valley the European granary^48^. In Egypt, the concept of perennial irrigation was known^49^. In India, (Bharat), Irrigation—as a powerful action against drought—combated famine, mitigated horrors, prevented terrible disasters, and protected lives^50^. Since ancient times, the additional value of irrigation as a means to optimize the productive efficiency of every water drop has been investigated examples included fertilizing soil^51^, intensifying cultivation, and fruiting arable land^52^, maintaining crops productivity by reducing soil alkalinity, and salts concentration^53^, economizing water in arid regions^54^, inhibiting Smut Diseases^55^, maximizing the fruit and seed yield at flowering^56^, and determining seawater irrigation impact on soil microflora^57^. Recently, on the other side, ecologists and environmentalists have combated the execution of irrigation plans without considering possible environmental impacts generated by the irrigation scheme^58^, environmental impacts could include for example, aggravating waterlogging, and water-retentive soil areas, decreasing crop yields, submerging archaeological sites, and resettling displaced people^59^.

The necessity of sustainable irrigation was emphasized by many scholars (e.g.,^60^). Surface water include rivers, streams, water courses, channels … etc. Groundwater includes aquifers, springs, wells … etc. Groundwater depletion accelerates globally, [e.g., the world,^61^, United States,^62^, India,^63^, Pakistan,^64^, South Asia,^65^]. Conjunctive surface and groundwater management could build a resilient water future^66^. The global hydrological model (GHM) has been employed to assess irrigation scenarios for bioenergy crop plantations^67^. In the United States, sustainable cropping systems have been proposed based on the irrigation scheme which is plant-centric^68,68^.

Sustainable agriculture has recently acquired more attention. A structural equation model (SEM) has been utilized to assess how aging threatens sustainable farming in China^69^. An agricultural nitrogen (N) blueprint to maximize agricultural yields, food sufficiency, ecological performance, and environmental sustainability and minimize nitrogen (N) consumption has been established^70^.

### Irrigation equilibrium indicators

In designing irrigation equilibrium indicators for a sustainable irrigated agriculture (SIA) project with its various components, it is important to distinguish between various kinds of indicators. In designing irrigation equilibrium indicators, one should give due attention to the project's time factor. Are the performance indicators going to be applied during the project execution period or they will be used after the project completion? In other words, which of these questions we are trying to answer through the usage of the indicators: Is progress happening? or did it happen? This is a significant factor to avoid traps in selecting suitable indicators since some activities need a long time to materialize, especially those about institutional changes.

Identifying correct performance variables is a complex process that must have input from various disciplines concerned with irrigated agriculture. The main set of guidelines for analyzing actual performance concern the identification of performance indicators and variables for evaluating irrigation scheme performance. Each component of the climate, water, food, and energy (CWFE) nexus would have specific objectives and an array of required outputs. Each component should somehow strengthen the overall objectives (OGs) of the sustainable irrigated agriculture (SIA) project. Indicators that are relevant to measure the performance of a specific component are designated micro indicators while those pertaining to the overall project objectives are designated macro indicators. Too many indicators could cause concentration loss and may diffuse issues. In designing performance indicators, one should be careful not to build traps. For instance, the sustainability of irrigation water users’ associations as an indicator is as difficult to measure as marriage sustainability. It takes a very long time to develop sustainable water user associations.

Ideally, the indicators should be cost-effective and quantitative where numbers are assigned to measure the performance level objectively. Performance indicators could be used by others to produce the same or similar results. Sometimes it is very difficult to arrive at quantitative indicators and qualitative indicators are the maximum that can be achieved. Indicators are needed that ideally meet the following criteria: few, have validity, can show an effect, can be objectively measured, and data needed could be collected quickly and efficiently. Equilibrium indicators could measure the magnitude of outputs to be compared to a desired predetermined level to reflect the climate, water, food, and energy (CWFE) nexus’s success in meeting its objectives. The effectiveness of output regarding its relevance and quality is also important.

### Unconventional water resources and Irrigation efficiency

The main thrust of the climate, water, food, and energy (CWFE) nexus activities on the Sustainable irrigated agriculture (SIA) is to strengthen the overall reliability. When utilizing reuse of irrigation scheme loss (seepage, deep percolation, runoff … etc.); and when considering unconventional water resources techniques (reuse of domestic wastewater, agricultural drainage …etc.), the overall irrigation scheme efficiency in country like Egypt could be about 95%. In the case of quite high irrigation scheme efficiency, opportunities for conserving freshwater by enhancing the irrigation efficiency at the local level have little room to be optimized. In that case, climate, water, food, and energy (CWFE) nexus activities on the farm level focus on improving the system flexibility to allow for better scheduling and improving system control to give tailenders a fair chance to utilize their adequate share of water.

The effect of irrigation improvement activities on both the quantity of drainage water and on the underlying groundwater conditions should be studied to be able to address quantitively the effect of localized improvement activities on the overall water balance and the integrated system efficiency. It is important to assess and monitor the performance of each individual component and see that the nexus is achieving its goals.

In the case of agricultural civilizations like Egypt, using traditional irrigation schemes could already produce fruitful yields at a very high level. Therefore, equilibrium indicators could focus on the variation of yield along the mesqa, or along the supply canal rather than the yield in absolute terms. The increase in yield due to climate, water, food, and energy (CWFE) nexus components reflects the effects of better scheduling and improved system reliability and control. Yields at the tail ends of the mesqa or canal are anticipated to intensify and consequently, the overall production sustainably increases.

### The multidisciplinary approach and future perspective

The function and objectives of climate, water, food, and energy (CWFE) nexus components are related to intermeshing boundaries. Communication and interaction between these components should be investigated. Examples of areas of interaction are given. Training activities should be related to all climate, water, food, and energy (CWFE) nexus components, and concentrated on the overlap regions within the nexus. Roadmaps, tools, and models produced by futurology, planning studies, and mathematical models could be applied to enhance the operation of the irrigation delivery system. Strengthening role of the information technology, and artificial intelligence in the monitoring, evaluation, and performance measurement of climate, water, food, and energy (CWFE) nexus activities. A possible example is a close interaction between water allocation practice, irrigation schemes, and irrigation improvement activities. Close interaction between preventive maintenance, and hydraulic structures rehabilitation and replacement, to set optimal schedules of maintenance versus replacement.

The vision of artificial intelligence (AI) was highlighted in the context of water, agriculture, and sustainable development. Artificial intelligence (AI) computerizations would support the water, food, and energy sectors^71^. Responsible application of artificial intelligence in water systems (e.g., management, design, operation, maintenance, water justice, and emergency response, …) was emphasized^72^. Artificial intelligence (AI) and machine learning (ML) were suggested to enable sustainable precision agriculture by application of nanotechnology, nanomaterials, and nanoinformatics^73^. Biodiversity is a key physical contributor to people’s lives and ecosystem services (e.g. clean water, food, pollination, clothing, and medicine, and many other contributions …), usage of artificial intelligence and reinforcement learning to improve biodiversity protection was discussed^74^. Risks and reliability of artificial intelligence and machine learning in agriculture were discussed^75^.

Many studies could be recommended to measure the influence of the CWFE nexus on the performance of the irrigation scheme. Examples of such studies are given Monitoring, assessing, and analyzing the influence of irrigation efficiency improvement components on optimizing production and water control in the cultivable command areas (CCA). Integrated or individual impact of hydraulic structures rehabilitation and replacement, preventive maintenance, and artificial intelligence, on reliability and control of irrigation practices. Merits of using the advanced (climate, water, food, and energy) nexus research, futurology, and sustainability studies, to enhance the irrigation scheme performance over the current practices based on experience or rules of thumb.

Studies to answer the following fundamental question of "how to optimize irrigation scheme efficiency?" are still necessitated. Answering such a question would lead to an accurate estimate of the quantity of water that could be conserved for other uses. Equilibrium indicators outlined in this study would be beneficial in this regard. It is further required to validate and apply the proposed indicators to actual field conditions. This would necessitate collaboration among all components to obtain available data or take the necessary steps to monitor and collect the required data. Examples of studies that are necessitated to judge the impact and usefulness of the climate, water, food, and energy (CWFE) nexus are given: Monitoring and analyzing the irrigation improvement activities on the agricultural production, and water control in the command areas under improvement. Merits of using artificial intelligence s over current practices based on experience or rules of thumb. The integrated or individual impact of hydraulic structures rehabilitation and replacement, Preventive Maintenance, information technology, and artificial intelligence on the reliability and control of the main delivery system. Impact of training activities on job performance. The optimizing CWFE nexus is a pathway for comprehensive mechanism for sustainable irrigated agriculture (SIA). The optimization of the CWFE nexus is a logical pathway for a comprehensive mechanism for sustainable irrigated agriculture (SIA). The influence of the (CWFE nexus and its contributions to sustainable irrigated agriculture (SIA) performance and agricultural production could be a good path for future improvements.

The final concern is what could happen if there will not be sustainable finance. The question of sustainability becomes of prime importance. Careful attention should be given to providing means of budget availability, continued staff, and priority criteria for both activities and programs.

## Conclusions

Multidisciplinary, cross-sectoral, and trans-disciplinary approaches to develop innovative tools for effective water, food, and energy management in the context of climate-adaptive policies have been applied. Sustainable, smart, and inclusive development and wisely rationalizing utilization of natural resource treasures are vital for humanity, life, and sustainability. Sustainability of man-made and natural resources related to their ability to remain in business. The equilibrium state in the sustainability context could be comprehended as the balance, and adaptation of competing actions, divergent influences, and/or opposing forces, in both static and dynamic situations for all man-made and natural modules components, and elements. For the sustainability situation, the ultimate measure of equilibrium is sustainable productivity and yields, environmental balance, optimal economic returns, and social status development. The goal of the Irrigation equilibrium indicators (IEIs) could be stated as: “Effective control of surface water and groundwater for optimal climate adaptation and optimal allocation for agriculture, to maximize freshwater, agricultural, and energy, productivity”. For each development objective, equilibrium indicators exist that describe how well a scheme is meeting an objective. For example, equity, adequacy, and reliability of freshwater delivery can indicate how the water control purpose is being met. It is a must to analyze the system equilibrium to see whether the objectives are being achieved. This is done by comparing actual performance with potential or desired performance. The gap between actual and desired system performance begins to establish the priority problem areas where the performance is low. Contributing factors to the priority problem areas should be delineated. Recommendations are then formulated to establish goals to resolve these contributing factors. A rapid appraisal may not provide time and resources for measuring the scheme performance in attaining an objective, thus equilibrium indicators may provide a basis for understanding the status of scheme performance. Irrigation and yield efficiency could be good equilibrium indicators. The key equilibrium indicators could describe the status at the termination of the sustainable irrigated agriculture (SIA) project. Concerning water organizations, capabilities should be enhanced in all primary functions of planning, design, operation, and maintenance. Therefore, the technical goal is “enhancement of the operating freshwater distribution efficiency for agricultural irrigation and other water applications”. Hence, the Water organizations’ capabilities and capacities to plan—design—build—operate—maintain (PDBOM) would be accordingly strengthened. At the project component level, objectives are stated with the expected magnitude of output. The equilibrium indicators implemented by Water organizations could often necessitate strengthening advanced capabilities in planning, designing, operating, and maintaining the irrigation scheme for sustainable irrigated agriculture (SIA). Water organizations’ role in planning, design, operation, and maintenance should be strengthened as an urgent and pivotal opportunity to cope with global, regional, and national challenges. Their capabilities in the climate, water, food, and energy (CWFE) nexus should be reinforced to accelerate action toward global goals. To ensure sustainability, future studies, planning models, project preparation, information technology, advanced technologies, and system management components could provide effective tools and integrated services to water organizations for sustainable system operation and management. It is valuable to mention that more cooperation among different components paves the road for more positive impact achievements.

## Supplementary Information

Supplementary Information.

## Data Availability

The datasets used and/or analyzed are available from the author on reasonable request.
