# Supplementary material for: Multi-disciplinary strategy to optimize irrigation efficiency in irrigated agriculture
Source: Sci Rep. 2024 May 19;14:11433. doi: 10.1038/s41598-024-61372-0 (PMC11636869; doi:10.1038/s41598-024-61372-0)
Supplement: Supplementary file 1 — Supplementary Information. [file 41598_2024_61372_MOESM1_ESM.docx]

**Appendices**

**Appendix 1. Mathematical symbols**

|  | **Symbol** | **Mathematical meaning** |
| --- | --- | --- |
| 1 | I_a_ | The potential irrigated area |
| 2 | *i*^th^ | The *i*^th^ s segment of the irrigation scheme |
| 3 | *n* | The number of seasons observed |
| 4 | *R_a_* | The ratio of agricultural yield before and after Climate Action (CA) (P.U.) of area |
| 5 | R_e_ | The ratio of agricultural yield before and after (CA) (P.U.) of energy |
| 6 | *R_w_* | The ratio of agricultural yield before and after (CA) (P.U.) of freshwater |
| 7 | W_d_ | The Water quantity delivered and applied (P.U.) area |
| 8 | W_d_(*i*) | The quantity of water allocated into the *i*^th^ section |
| 9 | W_L_ | The water losses |
| 10 | W_L_(*i*) | The water losses within the section |
| 11 | W_r_ | The required Water quantity for beneficial use (P.U.) area |
| 12 | W_t_ | The total available water Quantities |
| 13 | *Y_a_* | The crop yield (agricultural output) (P.U.) of area |
| 14 | *Y_aa_* | The actual yield (P.U.) of area |
| 15 | *Y_aj_* | The yield (P.U.) of area for the *j*^th^ season |
| 16 | *Y_aw_* | The actual yield (P.U.) of freshwater |
| 17 | *Y_ea_* | The expected yield (P.U.) of area |
| 18 | *Y_ew_* | The expected yield (P.U.) of freshwater |
| 19 | *Y_w_* | The crop yield (agricultural output) (P.U.) of freshwater |
| 20 | η | Efficiency |
| 21 | η_I_ | Irrigation efficiency |
| 22 | η_sec_ | The section efficiency |

**Appendix 2. Acronyms, and Abbreviations**

|  | **Acronyms/Abbreviation** | **Words/phrases** |
| --- | --- | --- |
| 1 | CA | Climate Action |
| 2 | CC | Climate Change |
| 3 | CWFE nexus | Climate, Water, Food, and Energy nexus |
| 4 | EIA | Environmental Impact Assessment |
| 5 | GGs | Global Goals |
| 6 | GHM | Global Hydrological Model |
| 7 | HS | Hydraulic structure |
| 8 | IEIs | Irrigation Equilibrium Indicators |
| 9 | PU | per unit (PU) (PU or P.U.) - Per Unit (pu) |
| 10 | SEM | Structural Equation Model |
| 11 | SIA | Sustainable Irrigated Agriculture |
| 12 | WRs | Water Resources |
